# The transcriptional and mutational landscapes of lipid metabolism-related genes in colon cancer

**DOI:** 10.18632/oncotarget.23592

**Published:** 2017-12-21

**Authors:** Lara P. Fernández, Ricardo Ramos-Ruiz, Jesús Herranz, Roberto Martín-Hernández, Teodoro Vargas, Marta Mendiola, Laura Guerra, Guillermo Reglero, Jaime Feliu, Ana Ramírez de Molina

**Affiliations:** ^1^ Molecular Oncology Group, IMDEA Food Institute, CEI UAM + CSIC, Madrid, Spain; ^2^ Genomics Unit, Parque Científico de Madrid, Madrid, Spain; ^3^ Biostatistics and Bioinformatics Unit, IMDEA-Food Institute, CEI UAM+CSIC, Madrid, Spain; ^4^ Molecular Pathology Section, Institute of Medical and Molecular Genetics (INGEMM) La Paz University Hospital, Madrid, Spain; ^5^ Molecular Pathology and Therapeutic Targets Lab, IdiPAZ, La Paz University Hospital, Madrid, Spain; ^6^ CIBERONC CB16/12/00398, La Paz University Hospital, Madrid, Spain; ^7^ Pathology Department, IdiPAZ, La Paz University Hospital, Madrid, Spain; ^8^ Clinical Oncology Department, La Paz University Hospital, Madrid, Spain; ^9^ Translational Oncology Lab, IdiPAZ, La Paz University Hospital, Madrid, Spain

**Keywords:** colorectal cancer, prognosis, lipid metabolism, gene expression profile, genetic variations

## Abstract

Metabolic alterations encountered in tumors are well recognized and considered as a hallmark of cancer. In addition to Warburg Effect, epidemiological and experimental studies support the crucial role of lipid metabolism in colorectal cancer (CRC). The overexpression of four lipid metabolism-related genes (*ABCA1, ACSL1, AGPAT1* and *SCD* genes) has been proposed as prognostic marker of stage II CRC (ColoLipidGene signature).

In order to explore in depth the transcriptomic and genomic scenarios of *ABCA1*, *ACSL1*, *AGPAT1* and *SCD* genes, we performed a transcriptomic meta-analysis in more than one thousand CRC individuals. Additionally we analyzed their genomic coding sequence in 95 patients, to find variants that could orchestrate CRC prognosis.

We found that genetic variant rs3071, located on *SCD* gene, defines a 9.77% of stage II CRC patients with high risk of death. Moreover, individuals with upregulation of *ABCA1* and *AGPAT1* expression have an increased risk of CRC recurrence, independently of tumor stage.

*ABCA1* emerges as one of the main contributors to signature’s prognostic effect. Indeed, both high *ABCA1* expression and presence of tumoral genetic variants located in *ABCA1* coding region, seem to be associated with CRC risk of death. In addition the non-synonymous polymorphism rs2230808, located on *ABCA1*, is associated with gene expression. Patients carrying at least one copy of minor allele showed higher levels of *ABCA1* expression than patients carrying homozygous major allele.

This study broaden the prognostic value of *ABCA1, ACSL1, AGPAT1* and *SCD* genes, independently of CRC tumor stage, leading to future precision medicine approaches and “omics”-guided therapies.

## INTRODUCTION

Colorectal cancer (CRC) is a heterogeneous disease that relevantly contributes to cancer mortality and morbidity. Over the past decade, many efforts have been performed by the scientific community, in order to find CRC expression patterns that allow us an accurate disease stratification into different prognostic subgroups and/or in connection with response to therapies. Next-generation sequencing (NGS) technologies have also been contributing to a better understanding of the CRC development. Through a fully integrated view of the genetic and genomic changes, NGS studies have provide information about CRC etiology together with identification of driver mutations that mediate the carcinogenesis processes [1–4].

In 2015, the intrinsic heterogeneity and molecular complexity of CRC led several international research groups to share large-scale data in order to establish a new CRC classification based on an unbiased approach [5]. Four transcriptomic consensus molecular subtypes (CMS) of CRC were defined: CMS1 or microsatellite instability (MSI) immune subtype, CMS2 or canonical, CMS3 or metabolic and CMS4 or mesenchymal subtype. Specifically, metabolic subtype tumors, CMS3, have been characterized as those which harbor KRAS mutations, a mixed MSI status, low somatic copy number alterations (SCNA) and low CpG island methylator phenotype (CIMP). Furthermore, CMS3 tumors exhibit a prominent metabolic activation with a clear enrichment for multiple metabolism signatures, in connection with the presence of KRAS-activating mutations that have been described as inducing metabolic reprogramming [5].

Metabolic alterations encountered in tumors are frequently described, they are well recognized and considered as a hallmark of cancer [6]. Cancer cells adapt their metabolic capacities to efficiently supply their novel demands of growth, proliferation and survival. In addition to Warburg Effect, lipid metabolism has been raised as crucial in tumor development, necessary to satisfied tumor requirements of biomass and structural components [7].

In the context of lipid metabolism, we identified an expression signature: ColoLipidGene (*ABCA1* (ATP-Binding Cassette Subfamily-A Member), *ACSL1* (Acyl-CoA Synthetase 1), *AGPAT1* (1-Acylglycerol-3-Phosphate O-Acyltransferase 1) and *SCD* (Stearoyl-CoA-desaturase 1) genes) that predicts prognosis in CRC patients of stage II [8]. Furthermore, our results generated a demand of improving knowledge of genes implicated in lipid metabolism: their function, regulation and roles in CRC [9]. The recent availability of large databases of patients with CRC, allow us to explore in depth, the transcriptomic and genomic landscapes of ColoLipidGenes (Genes of ColoLipidGene signature) in all stages of CRC. Thereby, we performed a transcriptomic meta-analysis on stored data at Gene Expression Omnibus (GEO) repository. In addition, since passenger mutations could also be a marker for tumor aggressiveness and response to passenger-exacerbating therapies [10], we explored the genomic coding sequence of ColoLipidGenes in CRC patients in order to find sequence variants and/or passenger mutations that could orchestrate CRC prognosis.

Our results broaden the prognostic value of *ABCA1*, *ACSL1*, *AGPAT1* and *SCD* genes in all stages of CRC, leading to future precision medicine approaches and “omics”-guided therapies.

## RESULTS

### Expression of metabolism-related genes is associated with CRC prognosis: Results from meta-analysis

**Figure 1:**
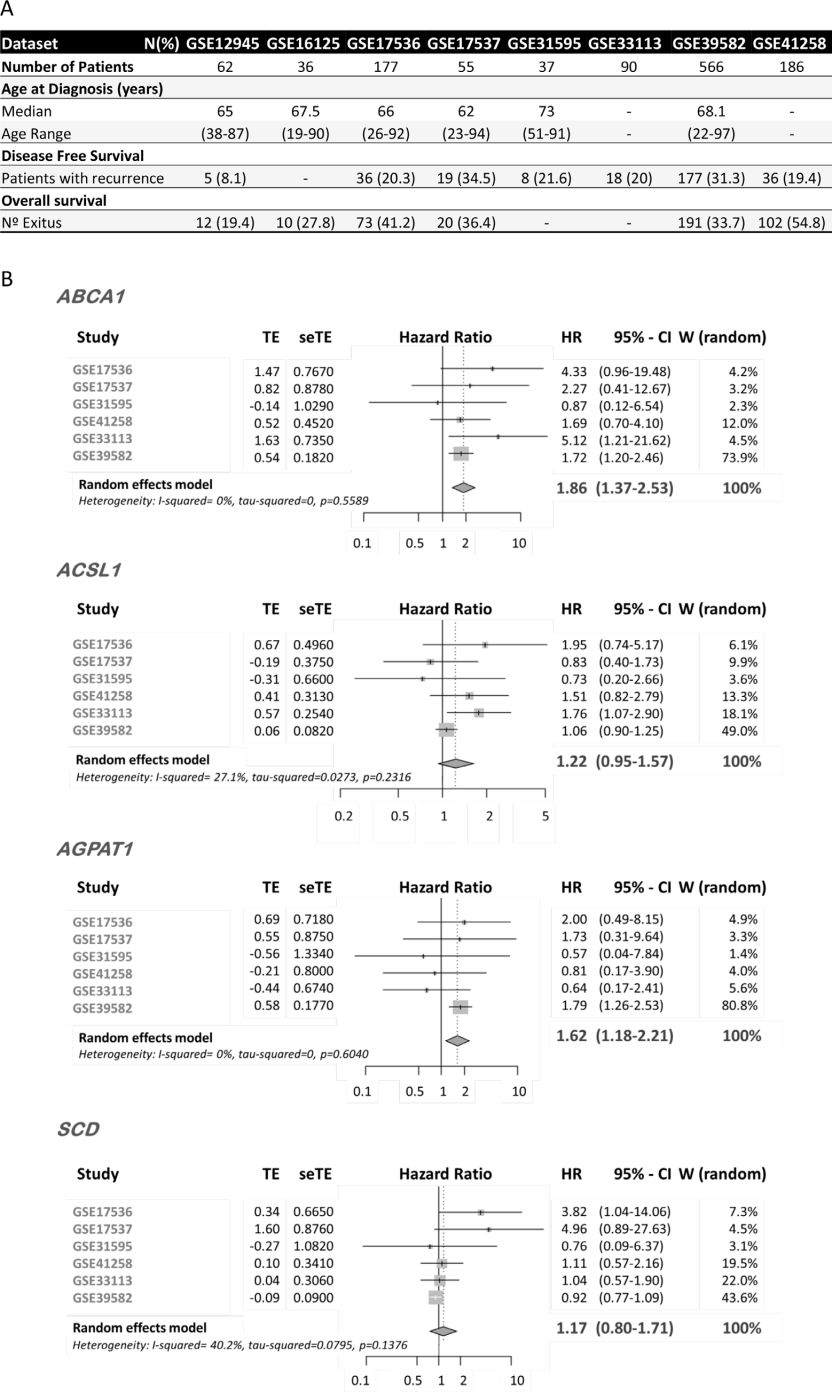
mRNA Expression of ColoLipidGenes and CRC prognosis (**A**) Clinical characteristics of GSE datasets included in the meta-analysis. (**B**) Forest plot showing the meta-analysis of hazard ratio (HR) and 95% confidence interval (CI) estimates for disease free survival (DFS) of lipid metabolism-related gene expression in CRC patients from six different studies. TE, seTE: Arcsine transformation of proportion and its standard error for individual studies. W (random): Weight of individual studies (in random effects model).

In order to test the prognostic value of ColoLipidGenes for patients with CRC tumors, we performed a meta-analysis based on publicly available transcriptomic data. We investigated whether the individual gene expression of *ABCA1*, *ACSL1*, *AGPAT1* and *SCD*, was associated with recurrence in a large series of CRC patients of stages from I to III, using gene expression datasets from GEO repository database. We selected six CRC datasets for which disease free survival (DFS) information was available (recurrence and time until recurrence in months), and with a minimum number of events (>5) (GSE17536, GSE17537, GSE31595, GSE41258, GSE33113, GSE39582). The meta-analysis comprised a total number of 1025 CRC cases (Figure 1A).

*ABCA1* and *AGPAT1* expression had an overall risk effect for recurrence in CRC patients, and the pooled Hazard Ratio (HR), was significantly higher than 1 (*ABCA1*, HR: 1.86 (CI 95%: 1.37–2.53) *p =* 0.0001, *AGPAT1*, HR: 1.62 (CI 95%: 1.18–2.21) *p =* 0.002) (Figure 1B). We detected a trend in association of *ACSL1* expression with overall risk effect for DFS (HR: 1.22 (CI 95%: 0.95–1.57) *p =* 0.1). Apparently, we are not able to detect an overall risk effect for *SCD* mRNA expression (HR: 1.17 (CI 95%: 0.8–1.71) *p =* 0.43169).

These data consistently validate our previous findings since, upregulation of *ABCA1*, *AGPAT1* and *ACSL1* is associated with worse outcome in CRC patients. Moreover, our data open up the use of these three genes as biomarkers of recurrence in all CRC stages.

### Catalog of somatic mutations in metabolism-related genes in colon cancer

**Figure 2:**
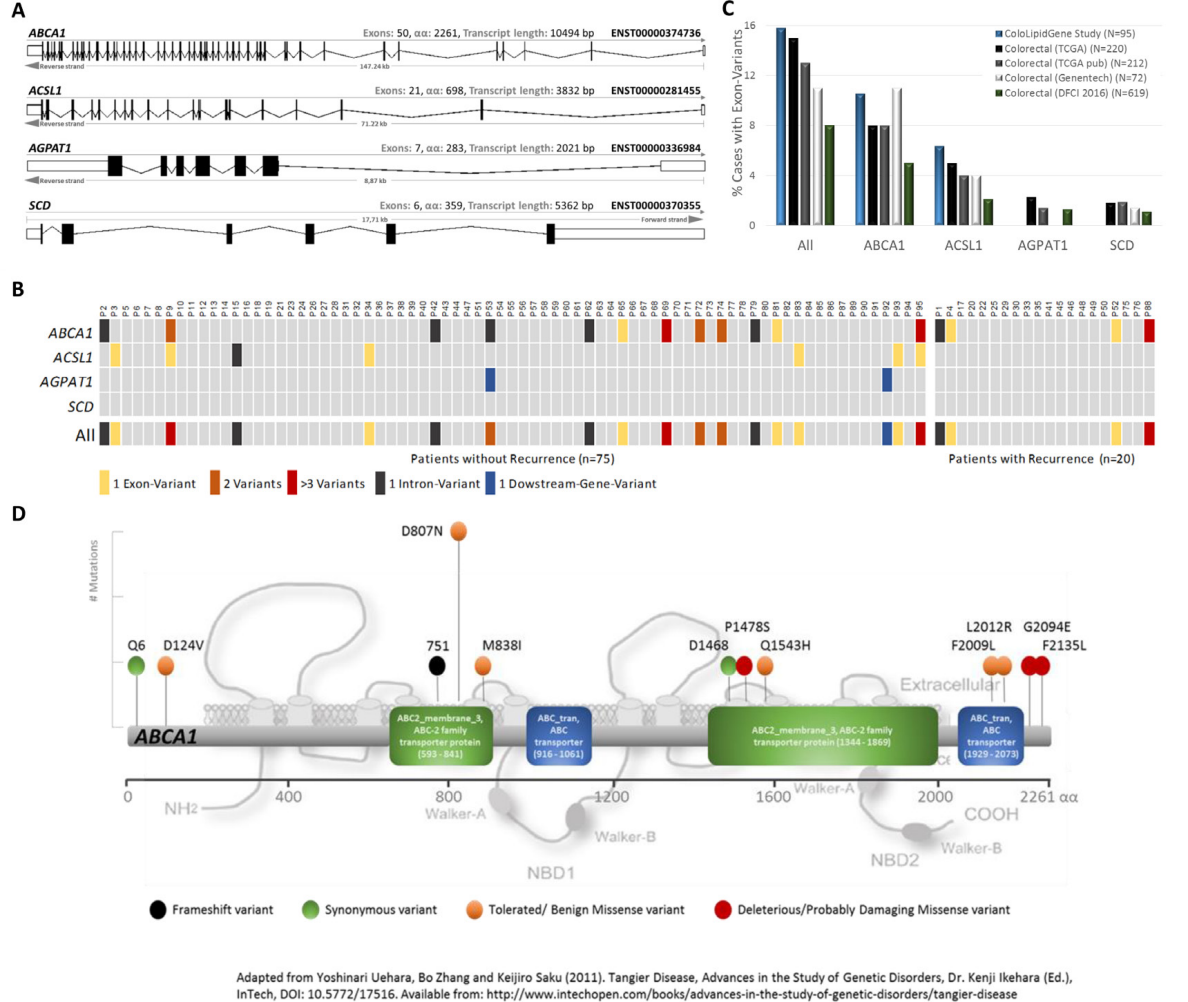
Variants of ColoLipid Genes found in colorectal cancer (CRC) patients (**A**) Graphic representation of sequenced metabolism-related genes belonging to ColoLipidGene study. (**B**) Oncoprint-type representation of gene variants found in this study. (**C**) Percentages of CRC patients with exonic variants found in ColoLipidGene study and in public-available databases. (**D**) Bi-dimensional representation of *ABCA1* gene and corresponding exonic variants found in 95 CRC patients from ColoLipidGene study. All represented variants have not been previously described.

We performed targeted re-sequencing of coding regions of ColoLipidGenes (Figure 2A) to determine type and number of metabolic-genes changes in CRC and to asses for their prevalence in a set of 95 stage II CRC patients (Table 1). Following sequential filtering and validating the next generation sequencing (NGS) approach with previous genotyping results (see Materials & Methods and Supplementary Table 1), we identified a total number of novel 25 single nucleotide variants (SNVs) mapping to three unique genes, *ABCA1*, *ACSL1* and *AGPAT1*. We did not find any SNVs in *SCD* gene (Figure 2B). We detected 15 (15.7%) CRC patients with single nucleotide variants (SNVs) mapping to exon regions of two unique genes, *ABCA1* and *ACSL1*. We did not find any patient with SNVs in coding regions of *AGPAT1* or *SCD* genes (Figure 2C). The mutational rates that we obtained, were similar to those obtained in publicly available databases [3, 4, 11] (Figure 2C). Type and frequency of SNVs found in *ABCA1* gene were detail in Figure 2D. We detected twelve SNVs in coding regions of *ABCA1* gene. Among them, Asp807Asn variant was found in three patients. Three of twelve SNVs were predicted to be deleterious in nature by SIFT and/or Polyphen algorithms [12, 13] (Figure 2D).

**Table 1:** Clinical characteristics of colorectal cancer patients from ColoLipidGene study (training group)

Characteristics	*N* (%)
**Patients**	95 (100)
**Age at Diagnosis (years)**	
Mean	65.23
Median	66
Age Range	26–86
Under 50	7 (7.4)
50–70	62 (65.3)
Over 70	26 (27.4)
**Gender**	
Female	39 (41.1)
Male	56 (58.9)
**Stage**	
IIA (T3 N0 M0)	66 (69.5)
IIB (T4 N0 M0)	29 (30.5)
**Primary Tumor Location**	
Cecum and Ileocecal Valve	9 (9.5)
Acending colon and Hepatic flexure	26 (27.4)
Transverse colon	8 (8.4)
Splenic flexure and Descending colon	9 (9.5)
Sigmoid colon and rectosigmoid junction	42 (44.2)
Rectum	1 (1.1)
**Grade/Differentiation**	
Well	7 (7.4)
Moderately	81 (85.3)
Poor	7 (7.4)
**Vascular Invasion**	
No	64 (67.3)
Yes	30 (31.6)
**Perineural Invasion**	
No	73 (76.8)
Yes	21 (22.1)
**Disease Free Survival**	
Patients with recurrence	20 (21.1)
**Overall survival**	
N° Exitus	11 (11.6)

### Associations of single nucleotide polymorphisms (SNPs) found in metabolism-related genes with colon cancer prognosis

**Figure 3:**
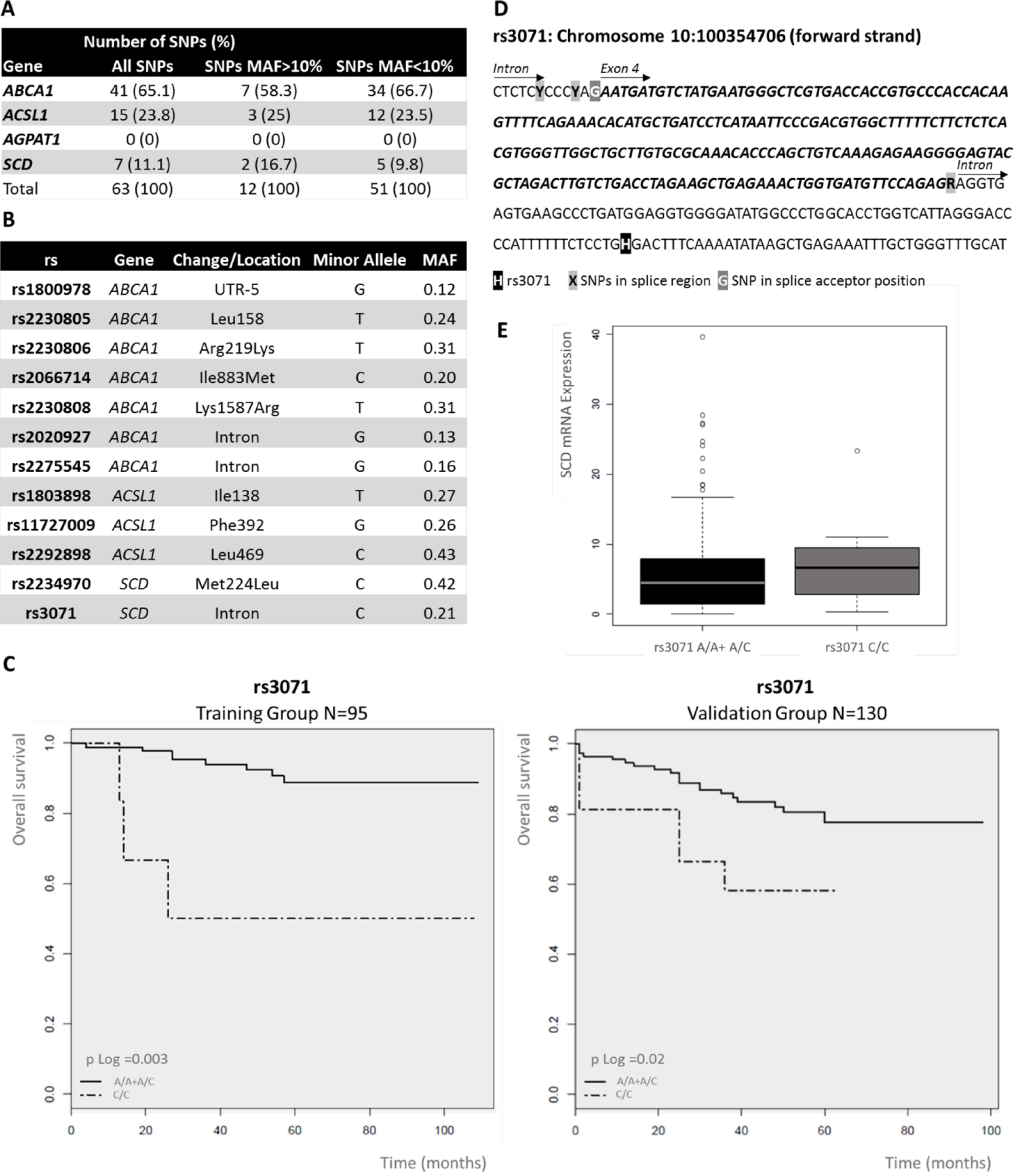
Analysis of single nucleotide polymorphisms (SNPs) of ColoLipidGenes: Prognostic value of rs3071 in stage II colorectal cancer (CRC) patients (**A**) Number and frequency classification of SNPs investigated in 95 stage II CRC patients. (**B**) Location, type and minor allele frequency (MAF) of polymorphisms, with MAF>10%, analyzed in the study. (**C**) Kaplan–Meier curves of *SCD* rs3071, on OS for stage II CRC patients in a recessive model of inheritance. Common/mayor Homozygote plus Heterozygote alleles (A/A + A/C); Variant/ minor Homozygote allele (C/C). *P*-value was calculated by Log-rank test. (**D**) Genomic context of rs3071. rs3071 is located in an *SCD i*ntron, next to a splice region. (**E**) Box plot of the association between gene expression level for *SCD* and genotype for rs3071. The box plot show how the *SCD* expression values are distributed for each genotype from recessive model of inheritance in all available stage II CRC patients (Training and Validation groups). Common/mayor Homozygote plus Heterozygote alleles (A/A + A/C); HV, Variant/ minor Homozygote allele (C/C).

Additionally, NGS approach allowed us to obtain information related to genetic polymorphisms of *ABCA1*, *ACSL1*, *AGPAT1* and *SCD* genes. We found a total of 63 SNPs (Figure 3A), 12 of them were relatively frequent with minor allele frequency (MAF) major than 10% and 51 of them were infrequent with MAF minor than 10%. We did not found any SNPs in *AGPAT1* gene. Then, we tested associations for metabolism-related SNPs with stage II CRC prognosis. We examined the impact on overall survival (OS) of 12 SNPs with MAF > 10% (Figure 3B). We found that two genetic variants were associated with clinical outcome of the patients, both in *SCD* gene (rs223490 and rs3071). Thus, C/- genotype for rs2234970 in the dominant model of inheritance (HR 0.11; 95% CI 0.02–0.45; *p*-value = 0.009) and C/C genotype for rs3071 in the recessive model (HR 12.3; 95% CI 2.78–54.38; *p =* 0.05) were significantly associated with the clinical outcome of CRC patients. The Kaplan–Meier survival curves and the log-rank test also showed the association between OS and rs2234970 (*p =* 0.01) and rs3071 (*p =* 0.003). In order to confirm the association of these two SNPs in *SCD* gene with the clinical outcome of CRC patients, we genotyped them in an independent validation set of 130 stage II CRC patients (validation group). Clinical characteristics of validation group are summarized in Supplementary Table 2. SNPs analysis in the validation group confirmed the potential prognostic value of rs3071 (HR 6.3; 95% CI 2.13–18.61; *p =* 0.003) (Figure 3C). We were not able to replicate in the validation group, the effect of rs2234970 on CRC prognosis (data not shown). The Kaplan–Meier survival curve and the log-rank test also confirmed the association between OS and rs3071 (*p =* 0.01) (Figure 3C). Thus, *SCD* genetic variant rs3071 defines a 9.77% of C/C CRC stage II patients with high risk of death. All *p*-values shown are adjusted for potential confounding factors and corrected for multiple comparisons by Bonferroni test.

The SNP rs3071 is located in proximal intron region of *SCD* gene next to a predicted splice region (Figure 3D). *SCD* is composed by 6 exons (Figure 2A). Only one transcript variant for *SCD* gene have been described (ENST00000370355). We assessed whether rs3071 polymorphism was associated with SCD gene expression in the complete series of patients (training and validation groups). We observed that patients carrying homozygote C/C alleles show a trend to have higher levels of SCD mRNA expression than the others. However, as expected, due to low frequency of minor homozygote C/C, results showed no statistically significant correlation between rs3071 genotype and *SCD* gene expression (Figure 3E).

### Genetic variation and gene expression of *ABCA1* modulate the outcome of CRC patients

**Figure 4:**
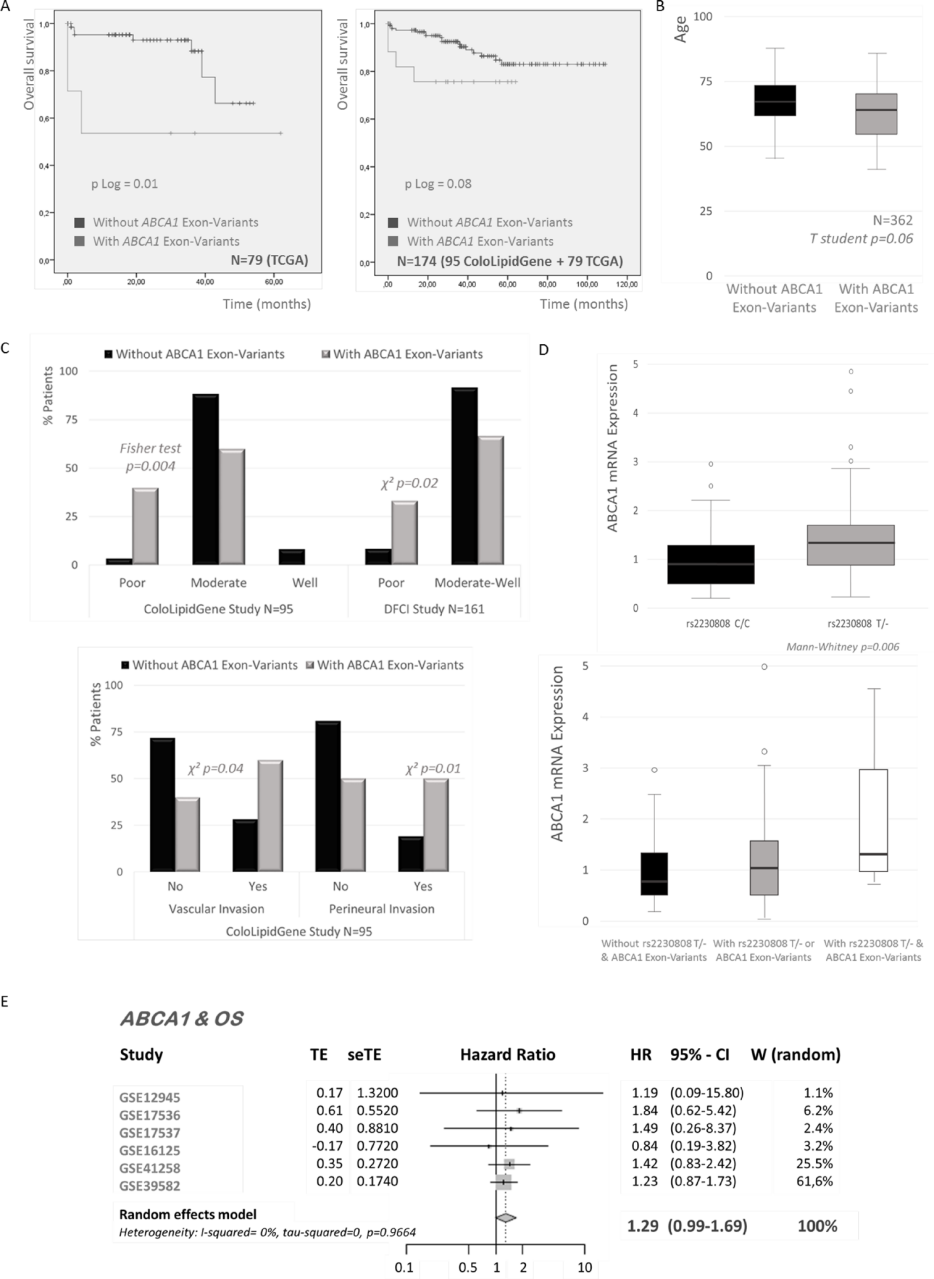
Associations of *ABCA1* variants and colorectal cancer (CRC) (**A**) Kaplan–Meier plots of *ABCA1* exonic variants and CRC overall survival (OS). Data from two populations (ColoLipidGene and TCGA studies) of Stage II CRC patients are used. P Log Rank values are shown. (**B**) Association of exonic variants in *ABCA1* gene and age of CRC diagnosis in 362 Stage II patients of three populations (ColoLipidGene, TCGA and DFSI studies). Student’s *t*-test was applied to assess statistically significant differences. (**C**) Associations of exonic variants in *ABCA1* gene and tumor grade, vascular invasion and perineural invasion of CRC in Stage II patients of two datasets (ColoLipidGene and DFSI studies). Student’s *t*-test or Fisher´s exact test were applied to assess statistically significant differences. (**D**) Associations of rs2230808 T allele and/or *ABCA1* exonic variants with *ABCA1* levels of expression. The Mann–Whitney *U*-test was used to compare differences between two groups and the Kruskal–Wallis test for comparison of three groups. (**E**) Forest plot showing the meta-analysis of hazard ratio (HR) and 95% confidence interval (CI) estimates for OS of *ABCA1* gene expression in CRC patients from six different studies.

Notably, *ABCA1* gene displayed the highest variation rate per sequenced base among ColoLipidGenes (SNVs/base in *ABCA1* = 0.003, *ACSL1* = 0.002, *AGPAT1* = 0.001 and *SCD* = 0). Type and frequency of SNVs found in *ABCA1* coding region were illustrated in Figure 2D. We studied the relationship between the presence of *ABCA1* variants and the CRC outcome. Probably due to low sample size and low number of genetic changes, we were not able to detect any statistically significant association (data not shown). Additionally, *ABCA1* sequence data from 79 patients of TCGA (The Cancer Genome Atlas) study, were interrogated for associations with CRC prognosis. We observed that, CRC patients carrying at least, one mutation in exonic sequence of *ABCA1* gene displayed poor overall survival (*p =* 0.01) (Figure 4A). In order to increase sample size, we pooled data from ColoLipidGene study (*N =* 95) together with TCGA study (*N =* 79) and we analyzed associations of SNVs in *ABCA1* coding region with outcome in 174 CRC patients. We observed a trend (*p =* 0.08) in the association of tumoral genetic variants located in *ABCA1* coding region with CRC overall survival (Figure 4A).

Next, we assessed for associations of SNVs in coding region of *ABCA1* with clinical and/or tumoral characteristics. We used data from ColoLipidGene study and two publicly available datasets (TCGA and DFCI (Dana-Farber Cancer Institute)) with the purpose of obtaining the maximum sample size. We detected that the presence of exonic variants of *ABCA1* were marginally associated to young patients (*p =* 0.06) in the pooled dataset (*N =* 362) (Figure 4B). Moreover, we observed several associations with prognostic clinical characteristics such differentiation grade and tumors with vascular and perineural invasion (*p* > 0.05) (Figure 4C).

Likewise, we also analyzed in ColoLipidGene study, the effect of SNPs in *ABCA1* gene on CRC development. We found that non-synonymous SNP, rs2230808 (Lys1587Arg), was associated with *ABCA1* gene expression (*p =* 0.006). Patients carrying at least one copy of minor allele T showed higher levels of *ABCA1* expression than patients carrying homozygous major allele (C/C) (Figure 4D). Moreover, we assessed the effect of rs2230808 together with the presence of SNVs on *ABCA1* expression levels. We observed that individuals carrying at least one copy of T allele in rs2230808 together with SNVs in coding region of *ABCA1* showed higher *ABCA1* mRNA expression than those carrying any of the two genetic changes separately (Figure 4D).

Finally, we tested by meta-analysis, whether the individual gene expression of *ABCA1* was associated with death in 1073 CRC patients of stages from I to IV, using six gene expression datasets from GEO repository database (GSE12945, GSE16125, GSE17536, GSE17537, GSE39582, GSE41258) (Figure 1A), for which OS information was available. *ABCA1* expression had an overall risk effect for OS in CRC patients, and the pooled HR was 1.29 ((CI 95%: 0.99–1.69) *p =* 0.06) (Figure 4E).

Collectively, these results indicate that gene expression together with genetic variation of *ABCA1* orchestrate CRC prognosis, being *ABCA1*, one of the main contributors of ColoLipidGene signature’s prognostic value.

## DISCUSSION

Metabolic signatures have acquired substantial relevance in the management and classification of CRC since the consensus molecular subtype CMS3 was recently redefined. CMS3 tumors exhibit a prominent metabolic activation with a clear enrichment for multiple metabolism signatures [5]. In this study, a comprehensive genomic analysis of genes belonging to ColoLipidGene signature [8] was performed. The genomic coding sequence of *ABCA1*, *ACSL1*, *AGPAT1* and *SCD* genes, as well as, their differential expression in a genome-wide expression meta-analysis in colorectal cancer patients were explored.

Due to recent develop of high throughput data techniques, nowadays, we have the possibility to use data publicly deposit in big repositories [14]. ColoLipidGene signature was defined from a limited group of lipid-related genes in stage II CRC patients [8]. We extended the prognostic value of ColoLipidGenes to large-scale micro-array profiling studies that include all stages of CRC cases. Several CRC datasets were needed to be comparable for meta-analysis, which required a standardized annotation. Two common problems of publicly available genomic data are the scarcity of clinical annotation and inconsistent definitions of clinical characteristics across independent datasets [13]. We reviewed original papers and performed curation of clinical annotations. We were able to retain, in several studies, the clinical variables of proven importance: disease free survival and overall survival (Figure 1A).

Our study, provides a comprehensive, highly curated and efficient CRC meta-analysis of ColoLipidGenes. The important use of this meta-analysis is the assessment of ColoLipidGenes as prognostic biomarkers in all stages of CRC. We have validated ColoLipidGene signature by analyzing individual gene contribution in a huge population of more than one thousand CRC patients from six different studies and representing tumors of all grades (Figure 1B). Among ColoLipidGenes, uniquely, we were not able to replicate *SCD* individual effect. We hypothesized that *SCD* action could be compensated for *ABCA1* and *AGPAT1* effect. Interactions among ColoLipidGenes have been previously described [15–18] by *in vitro* and bioinformatics approaches, that predict a well-established network of action (Supplementary Figure 1). Unequivocally, the *ABCA1* and *AGPAT1* expression seem to be important contributors to the ColoLipidGene signature prognostic value.

We explored genomic coding sequence of ColoLipidGenes in order to find passenger mutations that could be modulating prognosis, recurrence or outcome [10, 19]. It have been hypothesized a unique framework for understanding cancer progression as a balance of driver and passenger mutations [19]. Several studies suggested that mildly-deleterious passengers accumulate, collectively slow cancer progression, reduce the fitness of cancer cells and enhance the effects of therapeutics. We identified 15 (15.7%) CRC patients with SNVs mapping to exonic regions of two unique genes, *ABCA1* and *ACSL1* (Figure 2B). The *ABCA1* gene have the highest mutation rate that was in the same line which that reported in previous studies, TCGA [3], Genentech [4] and DFCI [11] (Figure 2C). Data from these studies were available in cBioPortal database [20, 21] and occasionally, they allowed us to increase our sample size.

We detected several genetic changes in *ABCA1* gene that could be modulating CRC prognosis (Figure 2D). These passenger mutations could be implicated in many not yet known aspects of carcinogenesis and tumor development. We found that exonic variants of *ABCA1* gene could be associated with CRC outcome as well as age of tumor development, tumor grade, vascular invasion and perineural invasion (Figure 4A–4D). We recognize that the sample size of this study was relatively limited and we simply lacked power to detect other associations.

In addition to mutation and gene expression analysis, we also performed single nucleotide polymorphisms (SNPs) susceptibility analysis from sequencing data. SNPs alteration is the most common genetic variation in the human genome [17]. SNPs in metabolic genes and their association with CRC prognosis have been previously evaluated in our population but only for recurrence detection [22]. Here we described novel associations with tumor characteristics and with death event.

We were able to validate SNP rs2230808 effect on *ABCA1* expression [23–25]. Moreover, we detected a clear association of *SCD* SNP rs3071 with death event in both training and validation groups (Figure 3C). Consequently, rs3071 allow us the identification of a 9.77% of CRC patients in risk. The SNP rs3071 is located in proximal intron region of *SCD* gene next to a predicted splice region. Moreover, rs3071 has been associated with cardiometabolic risk factors since its presence modified IL6 expression [23]. *SCD* gene seems to modulate CRC outcome at genomic level though rs3071 and at transcriptional level by interactions with ColoLipidGenes, specially *ABCA1* and *AGPAT1* [15–18] (Supplementary Figure 1).

Probably due to lack of statistical power, we were not able to detect any genetic change in exonic regions of *AGPAT1* or any genetic association with risk in *ACSL1* gene. On the contrary, the meta-analysis showed that the *ACSL1* levels of expression and specially those of *AGPAT1* clearly correlated with recurrence in all CRC patients (Figure 1B).

Finally, our results suggested that *ABCA1* gene is the most important contributor to prognostic value of ColoLipidGene metabolic signature probably though a combination of transcriptomic and genetic effects. *ABCA1* has emerged as the major cellular cholesterol efflux transporter, which has been implicated in several diseases like atherosclerosis, obesity and cancer [24–26]. The *ABCA1* expression is clearly associated with two indicators of CRC outcome: DFS and OS, in patients of all tumor stages (Figure 1B and Figure 4E). Moreover, we detected several genetic changes of *ABCA1* that could be also orchestrating prognosis. Passenger mutations in exonic regions of *ABCA1* could be implicated in many not yet known aspects of carcinogenesis. Likewise SNPs located in *ABCA1* are influencing *ABCA1* expression and consequently they could be implicated in CRC prognosis.

*ABCA1* alterations could have a role in the transition among tumoral stages that must be further explored. We could hypothesize that responsible mechanisms will include cholesterol transport alterations, specific tumor microenvironment interactions and/or the acquisition of tumor aggressiveness cell properties.

We have comprehensively characterized the mutational and transcriptional landscapes of ColoLipidGenes: *ABCA1*, *ACSL1*, *AGPAT1* and *SCD* in CRC. In the bases of precision medicine, it is presumed that genomic and transcriptomic data could be used for disease prognostication to stratify patients with CRC with different clinical outcomes. The present analysis reinforce the relevance of the prognostic value of ColoLipidGenes. Moreover, this study highlights the utility of *ABCA1* and *AGPAT1* as prognosis biomarkers of recurrence in CRC, independently of tumor stage.

## MATERIALS AND METHODS

### Patients’ samples

95 stage II CRC patients undergoing surgery between 2000 and 2004 in La Paz University Hospital were enrolled in the ColoLipidGene initial study. Population, tumor characteristics and inclusion criteria were described previously [8]. We validated the results in different sets of patients. SNP’s effect validation was performed in 130 stage II CRC patients (validation group) recruited in different time period (between 2004 and 2008) from La Paz University Hospital (Madrid) [8]. For these two groups of patients, Formalin-Fixed, Paraffin-Embedded (FFPE) samples were obtained with the approval of the human research Ethics review Committee of the hospital involved (HULP-PI-1452). Clinico-histopathological data of patients were resumed in Table 1 and Supplementary Table 2. We also used population’s data extracted from public datasets which characteristics were summarized in Figure 1 and Supplementary Table 3.

### Genomic analysis of ColoLipidGenes

Genomic DNA from formalin fixated paraffin embedded (FFPE) tissue of the patients was extracted using standard methods (QIAamp DNA FFPE Tissue Kit, Qiagen, Hilden, Germany) [22]. All DNA samples were quantified using Quant-iT PicoGreen dsDNA reagent according to the manufacturer’s instructions (Life Technologies, Foster City, CA). One ng of each sample was run on the Agilent 2100 Bioanalyzer to assess fragmentation and sample quality.

### Library preparation & sequencing

An enrichment system targeting the coding regions of genes *ABCA1*, *ACSL1*, *AGPAT1* and *SCD* was designed using Design Studio platform (Illumina) for TSCA (TruSeq Custom Amplicon) tools. Since samples were of FFPE origin and DNA degradation was anticipated, a double design was prepared, each targeting a different DNA strand, to minimize false variation rates. Designs expanded 100% of the regions of interest; details of the design can be consulted in the supplementary data accompanying this article (Supplementary Tables 4 and 5).

DNA samples were incubated with each of these collections of probes according to the manufactures instructions. Briefly, forward oligonucleotides were bound to target DNAs, extended and ligated to downstream primers in each of the DNA strands. Afterwards, PCR was used to amplify the collection of amplicons of each sample and to barcode individual samples by using the extension present in the prepared forward and downstream oligonucleotides. Individual profiles of these constructions were checked using a Bioanalyzer 2100 (Agilent) and traces were also used to estimate DNA concentrations of each sample. PoolA and pool B from each sample corresponding to both TSCA designs were also pooled together. Finally, samples were equimolar aggregated, cleaned and titrated using qPCR (Kapa) prior to sequencing.

Sequencing was made using two MiSeq cartridges (v2, 300 cycles, pair ended2x150) run under standard system conditions (Illumina). Following DNA sequencing, reads were demultiplexed according to sample barcodes and quality filtered so that fastq files were prepared from each individual sample. On average, more than 200.000 reads were obtained for each of the designs which correspond to an estimated coverage higher than 1.000×. Sequences were mapped to human genome using the re-sequencing pipeline of Illumina Base Space hub and the manifest files provided (Illumina) were used to select the coordinates to call variant positions. Finally, variants were visualized using off-line Variant Studio (Illumina) and used as starting point for variant filtering.

After sequential filtering, we compared sequencing results of SNPs with previous genotyping results of these patients [22] in order to validate sequencing approach. We found more than 95% of concordant results (see Supplementary Table 1).

### Genotyping

130 CRC samples from validation group were genotyped for rs2234970 and rs3071 SNPs, both located in SCD gene, as it has been previously described [22].

### Expression analysis of ColoLipidGenes

We used RNeasy Mini Kit or RNeasy FFPE Kit (Qiagen, Germantown, MD, USA) following manufacturer’s conditions to obtain total RNA from FFPE tumor samples previously deparafinated. Gene expression analysis was performed as previously described in *Vargas et al.* [8].

### Statistical analyses

Raw microarray data was downloaded from GEO database. The Aroma-affymetrix R package was used to analyze large volumes of data in a memory-efficient manner. For each study, CEL files were background corrected and normalized locally applying the RMA algorithm.

We included six CRC datasets for which disease free survival (DFS) information was available (recurrence and time until recurrence in months), performed in Affymetrix HG-U133A or HG-U133_Plus_2 platforms and with a minimum number of events (>5). R statistical software was used for efficient meta-analysis of ColoLipidGenes expression in CRC datasets [27].

SNVs were categorized as dichotomous (for carrying at least one variant, presence/absence) and SNPs were categorized by genotype (homozygote minor allele, heterozygote and homozygote major allele) and checked for additive, dominant and recessive model. Two-tailed Pearson and Fisher exact tests were used to compare distributions or allele frequencies. Bonferroni corrections for multiple comparisons were performed. In order to assess the prognostic value of genetic variants, presence of SNVs and genotypes of each polymorphism were tested for association with DFS and OS using univariate Cox-regression analysis, expressed as the hazard ratio (HR) with 95% confidence intervals (CI). To calculate the effect on survival with adjustment for potential confounding factors, proportional hazards Cox regression modeling was used including only variables that were significant (*p* < 0·05) in the univariate analysis. DFS was defined as the time from surgery until the first documented tumor recurrence or death. OS was defined as the time from surgery until death. The Kaplan–Meier method was used to estimate the survival probabilities, and the log-rank test was used to test differences between subgroups. Survival curves were illustrated according to the Kaplan–Meier method and the log-rank test was used to test for differences between the groups.

To evaluate the association between gene expression level and the different genotypes from the diverse models of inheritance, a non-parametric Kruskal–Wallis (KW) test and Analysis of Variance test (ANOVA) was performed. Expression data of genes (calculated with the 2–ΔCt method) were previously analyzed and reported in *Vargas et al.* [8].

All statistical calculations were carried out using the R statistical software version 2.15 (www.r-project.org). *P* values < 0.05 were considered significant, and all tests were two sided.

## SUPPLEMENTARY MATERIALS FIGURE AND TABLES









## Abbreviations

*ABCA1*: ATP-Binding Cassette Subfamily-A Member 1
*ACSL1*: Acyl-CoA Synthetase 1
*AGPAT1*: 1-Acylglycerol-3-Phosphate O-Acyltransferase 1
CI: Confidence Interval
CIM: CpG island methylator phenotype
CMS: Consensus Molecular Subtypes
CRC: Colorectal cancer
DFCI: Dana-Farber Cancer Institute
DFS: Disease Free Survival
FFPE: Formalin-Fixed, Paraffin-Embedded
GEO: Gene Expression Omnibus
HR: Hazard Ratio
MAF: Minor Allele Frequency
MSI: Microsatellite Instability
NGS: Next-generation Sequencing
OS: Overall Survival
*SCD*: Stearoyl-CoA-desaturase 1
SCNA: Somatic Copy Number Alterations
SIFT: Scale-invariant feature transform algorithm
SNP: Single Nucleotide Polymorphism
SNVs: Single Nucleotide Variants
TCGA: The Cancer Genome Atlas
TSCA: TruSeq Custom Amplicon
