# Supplementary material for: The transcriptional and mutational landscapes of lipid metabolism-related genes in colon cancer
Source: Oncotarget. 2017 Dec 21;9(5):5919–30. doi: 10.18632/oncotarget.23592 (PMC5814184; doi:10.18632/oncotarget.23592)
Supplement: Supplementary file 1 [file oncotarget-09-5919-s001.pdf]

The transcriptional and mutational landscapes of lipid metabolism-related genes in colon cancer

SUPPLEMENTARY MATERIALS

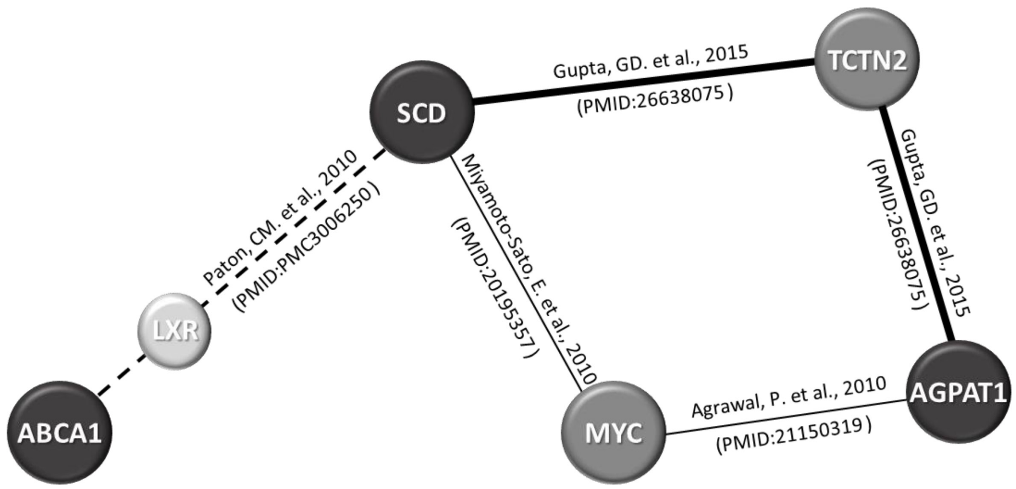

Supplementary Figure 1: Predicted interactome of *ABCA1*, *SCD* and *AGPAT1*. Interactions among *ABCA1*, *SCD* and *AGPAT1* have been previously reported in literature by in vitro and bioinformatics approaches.

Supplementary Table 1: Technical validations of next-generation sequencing (NGS) approach. See Supplementary_Table_1

Supplementary Table 2: Clinical characteristics of colorectal cancer patients from ColoLipidGene study (validation group)

**Characteristics**	***N* (%)**
**Patients**	130 (100)
**Age at Diagnosis (years)**	
Mean	70.31
Median	72
Age Range	96–23
Under 50	7
50–70	55
Over 70	68
**Gender**	
Female	59 (45.4)
Male	71 (54.6)
**Stage**	
IIA (T3 N0 M0)	80 (61.5)
IIB (T4 N0 M0)	50 (38.5)
**Vascular Invasion**	
No	103 (79.2)
Yes	26 (20.0)
**Perineural Invasion**	
No	102 (78.5)
Yes	27 (20.8)
**Disease Free Survival**	
Patients with recurrence	22 (16.9)
**Overall survival**	
N° Exitus	26 (20.0)

Supplementary Table 3: Clinical characteristics of colorectal cancer patients from TCGA and DFCI studies

**Dataset *N* (%)**	**Colorectal Adenocarcinoma (TCGA, Nature 2012)**	**Colorectal Adenocarcinoma (TCGA, Provisional)**	**lorectal Adenocarcinoma (DFCI, Cell Reports 2016)**
**Stage II Patients**	77 (100)	80 (100)	187 (100)
**Age at Diagnosis (years)**			
Mean	-	71.1	71.05
Median	-	72	71
Age Range	-	41–90	46–88
Under 50	-	4 (5)	1 (0.57)
50–70	-	34 (42.5)	86 (45.9)
Over 70	-	42 (52.5)	100 (53.5)
**Gender**			
Female	34 (44.1)	35 (43.7)	137 (73.3)
Male	43 (55.8)	45 (56.2)	50 (26.7)
**Disease Free Survival**			
Patients with recurrence	-	9 (11.2)	-
**Overall survival**			
N° Exitus	5 (6.5)	10 (12.5)	-
**Stage**			
IIA	73 (94.8)	76 (95)	-
IIB	4 (5.2)	4 (5)	-
**Grade/Differentiation**			
Well/Moderately	-	-	145 (77.5)
Poor	-	-	16 (8.5)

Supplementary Table 4: NGS Detailed designs for ColoLipidGenes I. See Supplementary_Table_4

Supplementary Table 5: NGS Detailed designs for ColoLipidGenes II. See Supplementary_Table_5
